# No association found between the detection of either xenotropic murine leukemia virus-related virus or polytropic murine leukemia virus and chronic fatigue syndrome in a blinded, multi-site, prospective study by the establishment and use of the SolveCFS BioBank

**DOI:** 10.1186/1756-0500-7-461

**Published:** 2014-08-04

**Authors:** David M Irlbeck, Suzanne D Vernon, K Kimberly McCleary, Lucinda Bateman, Nancy G Klimas, Charles W Lapp, Daniel L Peterson, James R Brown, Katja S Remlinger, David A Wilfret, Peter Gerondelis

**Affiliations:** 1 Division of Infectious Diseases, GlaxoSmithKline, Research Triangle Park, NC, USA; 2 The CFIDS Association of America, Charlotte, NC, USA; 3 Fatigue Consultation Clinic, Salt Lake City, UT, USA; 4 Nova Southeastern University College of Osteopathic Medicine, Institute for Neuro-Immune Medicine, Miami, FL, USA; 5 Hunter-Hopkins Center, Charlotte, NC, USA; 6 Sierra Internal Medicine Associates, Incline Village, NV, USA; 7 Department of Computational Biology, GlaxoSmithKline, Collegeville, PA, USA; 8 Statistical Consulting Group, GlaxoSmithKline, Research Triangle Park, NC, USA

**Keywords:** Chronic fatigue syndrome, Xenotropic murine virus-related virus, Polytropic murine leukemia virus, Retrovirus, PCR, Detection

## Abstract

**Background:**

In 2009, a retrospective study reported the detection of xenotropic murine leukemia virus-related virus (XMRV) in clinical isolates derived from individuals with chronic fatigue syndrome or myalgic encephalomyelitis (CFS). While many efforts to confirm this observation failed, one report detected polytropic murine leukemia virus (pMLV), instead of XMRV. In both studies, Polymerase Chain Reaction (PCR)-based methods were employed which could provide the basis for the development of a practical diagnostic tool. To confirm these studies, we hypothesized that the ability to detect these viruses will not only depend upon the technical details of the methods employed but also on the criteria used to diagnose CFS and the availability of well characterized clinical isolates.

**Methods:**

A repository of clinical isolates from geographically distinct sites was generated by the collection of fresh blood samples from well characterized CFS and healthy subjects. Molecular techniques were used to generate assay positive controls and to determine the lower limit of detection (LLOD) for murine retroviral and Intracisternal A particle (Cell 12(4):963-72, 1977) detection methods.

**Results:**

We report the establishment of a repository of well-defined, clinical isolates from five, geographically distinct regions of the US, the comparative determination of the LLODs and validation efforts for the previously reported detection methods and the results of an effort to confirm the association of these retroviral signatures in isolates from individuals with CFS in a blinded, multi-site, prospective study. We detected various, murine retroviral DNA signatures but were unable to resolve a difference in the incidence of their detection between isolates from CFS (5/72; 6.7%) and healthy (2/37; 5.4%) subjects (Fisher’s Exact Test, p-value = 1). The observed sequences appeared to reflect the detection of endogenous murine retroviral DNA, which was not identical to either XMRV or pMLV.

**Conclusions:**

We were unable to confirm a previously reported association between the detection of XMRV or pMLV sequences and CFS in a prospective, multi-site study. Murine retroviral sequences were detected at a low frequency that did not differ between CFS and control subjects. The nature of these sequences appeared to reflect the detection of pre-existing, endogenous, murine retroviral DNA in the PCR reagents employed.

## Background

The study of CFS suffers from a lack of (i) validated biomarkers to easily identify the condition and (ii) readily available, well-defined clinical isolates. Evidence for the detection of murine leukemia virus (MLV)-related viruses, including XMRV, in isolates derived from patients with CFS has been reported [1,2]. The implications of these findings for patients and the safety of the blood supply generated a global response from academic, regulatory and private institutions.

Confirmation of these studies would provide, at the least, a much needed tool to ease the huge burden associated with the diagnosis of this disease and enable clinical trials for the discovery of new treatments. In the period that followed the original reports, many studies failed to support a link between CFS and these viruses [3-24].

In this study we also endeavored to confirm a link between CFS and these viruses, but in a prospective manner with fresh, well-defined clinical isolates. To enable this effort, we carefully evaluated the methodologies of the original studies and established the SolveCFS BioBank (SCB); a patient-centered, advocacy-operated, repository of well-defined clinical isolates from geographically distinct regions of the United States. In this blinded, multi-site, prospective study we were able to detect sequences related to these viruses. However, we were unable to confirm a difference in their occurrence between specimens from CFS and healthy subjects. While we were unsuccessful in confirming the proposed link, this effort demonstrates the effectiveness of public/private partnerships in conducting studies of national scope. Further, the establishment and initial characterization of the SCB should prove to be a valuable reference and tool for the future discovery of CFS-related biomarkers.

## Results

### Study purpose, design and the establishment of the SolveCFS Biobank

To confirm reports of an association between CFS and the detection of murine-related retroviruses, the Chronic Fatigue and Immune Dysfunction Syndrome (CFIDS) Association of America, a group of CFS clinical sites and GlaxoSmithKline (GSK) partnered to conduct a blinded, multisite, prospective study with well-defined clinical isolates from a patient advocacy-centered, biological repository established for this and future studies. The SCB clinical isolate repository was successfully established with the collection of blood samples from 240 CFS and 87 healthy subjects from June 2010 to August 2010. Under the direction of the CFIDs Association coordinator, these isolates were procured and sent to the Rutgers University Cell and DNA Repository (RUCDR; Piscataway, NJ) for processing and storage. Matched health surveys and demographics were collected for each participant (Table 1).

**Table 1:** **Demographics**, **time of CFS**-**onset**, **physical health**, **mental health and clinical site for CFS**, **healthy and CFS positive control subjects**

		**CFS subjects**			**Healthy subjects**			**CFS positive control subjects**		
		**XMRV Positive (n** **=** **5)**	**XMRV Negative (n** **=** **67)**	**Total (n** **=** **72)**^**1**^	**XMRV Positive (n** **=** **2)**	**XMRV Negative (n** **=** **35)**	**Total (n** **=** **37)**^**1**^	**XMRV Positive (n** **=** **1)**	**XMRV Negative (n** **=** **19)**	**Total (n** **=** **20)**^**1**^
**Age (years), Mean (STDEV)**		50.4 (4.3)	49.4 (12.0)	49.5 (11.6)	30.0 (17.0)	47.6 (14.3)	46.6 (14.8)	28	51.4 (12.5)	50.0 (13.3)
**Age of first CFS symptoms (years), Mean (STDEV)**		30.6 (13.2)	34.8 (11.4)	34.5 (11.5)	NA	NA	NA	22	34.3 (12.3)	33.5 (12.2)
**Age of CFS diagnosis (years), Mean (STDEV)**		37.2 (11.3)	38.4 (11.3)	38.3 (11.2)	NA	NA	NA	24	37.1 (9.7)	36.3 (10.0)
**Sex**, **n (%)**	**Female**	4 (80)	51 (76)	55 (76)	2 (100)	29 (83)	31 (84)	1 (100)	9 (47)	10 (50)
	**Male**	1 (20)	15 (22)	16 (22)	0	5 (14)	5 (14)	0	7 (37)	7 (35)
	**Missing data**	0	1 (1)	1 (1)	0	1 (3)	1 (3)	0	3 (16)	3 (15)
**BMI (kg/m**^**2**^**) Mean (STDEV)**		26.2 (5.5)	25.9 (6.0)	25.9 (6.0)	21.2 (2.3)	25.6 (6.0)	25.3 (5.9)	18.6	24.6 (4.6)	24.2 (4.7)
**Race**, **n (%)**	**Caucasian**	5 (100)	66 (99)	71 (99)	2 (100)	33 (94)	35 (95)	1 (100)	15 (79)	16 (80)
	**Non**-**Caucasian**	0	0	0	0	0	0	0	1 (5)	1 (5)
	**Missing data**	0	1 (1)	1 (1)	0	2 (6)	2 (5)	0	3 (16)	3 (15)
**CFS Onset, n (%)**	**Gradual**	2 (40)	16 (24)	18 (25)	NA	NA	NA	0	8 (42)	8 (40)
	**Sudden**	3 (60)	50 (75)	53 (74)	NA	NA	NA	1	8 (42)	9 (45)
	**Missing data**	0	1 (1)	1 (1)	NA	NA	NA	0	3 (16)	3 (15)
**RAND**-**36 Physical Health Mean (STDEV)**	**Physical functioning**	20.0 (14.6)	36.3 (21.1)	35.1 (21.1)	100^2^	85.0 (30.2)	85.4 (29.8)	5	40.0 (28.3)	38.3 (28.6)
	**Role**-**physical**	20.0 (44.7)	2.3 (8.5)	3.5 (14.2)	50	90.7 (28.5)	89.6 (28.9)	0	1.3 (5.7)	1.3 (5.6)
	**Bodily pain**	48.0 (22.3)	47.7 (25.1)	47.7 (24.8)	90	84.4 (28.3)	84.6 (27.9)	30	43.8 (24.8)	43.1 (24.3)
	**General health**	24.0 (4.2)	24.9 (16.3)	24.9 (15.8)	90	76.1 (21.2)	76.5 (21.0)	10	15.3 (14.3)	15.0 (14.0)
**RAND**-**36 Mental Health Mean (STDEV)**	**Vitality**	12.0 (10.4)	14.7 (13.7)	14.5 (13.4)	55	68.6 (24.8)	68.2 (24.6)	10	17.9 (18.4)	17.5 (18.0)
	**Social functioning**	24.5 (14.5)	28.4 (22.2)	28.1 (21.7)	100	87.2 (26.7)	87.6 (26.4)	0	27.5 (28.1)	26.1 (28.0)
	**Role emotional**	86.7 (29.8)	67.2 (44.7)	68.5 (44.0)	100	86.7 (29.4)	87.0 (29.0)	100	56.1 (44.5)	58.3 (44.4)
	**Mental health**	67.2 (10.0)	60.7 (16.9)	61.2 (16.6)	68	76.1 (26.3)	75.9 (25.9)	64	53.5 (27.0)	54.0 (26.4)
**Clinical Site, n (%)**	**Florida**	0	6 (9)	6 (8)	0	14 (40)	14 (38)	0	0	0
	**North Carolina**	4 (80)	16 (24)	20 (28)	2 (100)	7 (20)	9 (24)	0	0	0
	**Pennsylvania**	0	1 (1)	1 (1)	0	0	0	0	0	0
	**Nevada**	0	0	0	0	0	0	1 (100)	19 (100)	20 (100)
	**Utah**	1 (20)	44 (66)	45 (63)	0	14 (40)	14 (38)	0	0	0

Data are derived from the RAND-36 Questionnaire and clinical site enrollment and separated into XMRV-positive and XMRV-negative. The majority of subjects were female and all subjects with race reported were Caucasian. Subject demographics between CFS subjects and Healthy subjects were similar. CFS subjects had lower physical health and mental health scores compared to Healthy subjects. Statistical comparisons between XMRV-positive and XMRV-negative categories were not feasible due to sample size limitations.

CFS: chronic fatigue syndrome; *XMRV*: xenotropic murine leukemia virus-related virus; STDEV: standard deviation; *MD*: missing data; *NA*: not applicable; *BMI*: body mass index: RAND-36: 36-item health-related quality of life questionnaire.

^1^Isolates from a 240 CFS (including 40 CFS Positive Control) and 87 healthy subjects were collected. Of these, 72 CFS, 20 CFS Positive Control and 37 healthy subjects were used in this study.

^2^Standard deviation not performed as RAND-36 data only available from one XMRV-positive healthy subject.

### Viral detection assay sensitivity comparisons and validation

During the effort to seed the SCB with biological specimens, several published methods were evaluated at GSK for use in this study. These methods involved the direct detection of viral DNA or RNA by PCR or RT-PCR, respectively. Positive controls for these assays were established to determine each assay’s LLOD. The sensitivity of the PCR-based methods for the detection of viral DNA were determined with a serial dilution of a molecular clone of the viral *gag* (pXMRV-Gag) prepared in a constant background of DNA purified from healthy, human peripheral blood mononuclear cells (PBMCs). By this approach, the LLOD of the PCR-based method to detect viral DNA described by Lombardi *et al*. [1] was found to be approximately 3 copies of viral DNA per 33,000 cell equivalents (200 ng). Similarly, the LLOD of the PCR-based method to detect viral DNA described by Lo *et al* (Lo Method) [2] was determined to be 5 copies of viral DNA per 8,250 cell equivalents (50 ng). We also tested the sensitivity of the method described by Lombardi *et al*. [1] to detect viral RNA by RT-PCR. The LLOD for this method was determined by the use of a serial dilution of purified *gag* RNA, prepared from *in vitro* transcripts by use of the T7 RNA polymerase promoter in pXMRV-Gag. Dilutions were prepared in a constant background of RNA purified from healthy, human PBMCs. By this method, the LLOD was found to be approximately 3 copies of viral RNA per 200 ng of total RNA. We also tested the sensitivity of this method using higher cell equivalent inputs, up to 2.5 μg per reaction. The LLOD increased as the input template RNA was increased (data not shown).

For the validation of methods for use in this blinded, prospective study, we procured a panel of clinical isolates from 20 individuals (CFS Positive Control Subjects), self-reported to have previously received a “XMRV-positive” test result by a third party. Application of the method for the detection of viral DNA described by Lombardi *et al*. [1] failed to identify any sequence of murine, retroviral DNA origin in these samples. However, application of the Lo Method did identify apparent, murine retroviral DNA in an isolate from one individual (G018) from this panel. Similarly, application of the method for the detection of viral RNA described by Lombardi *et al*. [1] resulted in the detection of murine, retroviral RNA in an isolate from one individual (G022) as well (data not shown). We also attempted a previously reported, pre-amplification by co-culture approach [25], however, we were unable to resolve any evidence of murine retroviruses by this method with plasma samples from the CFS Positive Control Subjects. Of the two methods in which we were able to detect murine, retroviral nucleic acid sequences neither appeared to provide an advantage in sensitivity (i.e. both detected 1 out of 20). As such, we elected to proceed with the Lo Method for use in our blinded, prospective, multi-site study as this method required the least amount of material per test which would allow for the execution of multiple technical replicates to assess reproducibility of results.

### Detection of murine retroviral DNA in both CFS and healthy subjects

Based upon initial reports of the apparent incidence of XMRV and pMLV [1,2], we estimated a need to attain fresh clinical isolates from at least fifty CFS patients with a CFS to healthy control ratio of about 2:1 (data not shown). After a sufficient number of isolates had been collected by the SCB and to begin to satisfy this estimate, an initial set of isolates from 109 individuals (72 CFS and 37 healthy subjects) were procured from the SCB for testing at GSK. The demographics, clinical onset of disease, degree of physical and mental health, and geographic region associated with these isolates are detailed in Table 1. Related statistical analyses are detailed in Additional file 1: Table S1 and Additional file 2: Table S2 as well as in Additional file 3: Figure S1. The demographics between the two study groups were similar (all p-values >0.19, Additional file 1: Table S1 and Additional file 2: Table S2), as almost all were derived from Caucasian individuals and most were female. However, with the exception of role emotional scale, physical and mental health scores reported by the CFS subjects were significantly lower than those reported by healthy subjects (Wilcoxon-Mann-Whitney test; p-values <0.0001).

With the operator blinded to the nature of these isolates, and by the use of the Lo Method, 5 of the 72 isolates derived from CFS subjects (6.9%) and 2 of the 37 isolates from healthy subjects (5.4%) resulted in a PCR positive and DNA sequence confirmed detection of a murine, retroviral nucleic acid signature. However, no significant difference in the prevalence of the detection of these signatures was observed between the CFS and healthy subject groups (Fisher’s Exact Test, p-value = 1).

Initial analyses to determine the nature of these sequences indicated that they were distinct from the VP62 and 22Rv1 reference sequences (Table 2) [26,27]. Since 22Rv1 was used to make the pXMRV-gag control, which was included in every test, the detected sequences did not arise from this source. In addition, PCR positive signals were not observed in the negative controls that were included in each test. Interestingly, a comparison of clones from each subject revealed some intra-subject diversity. Differences among these clones included coding changes in *gag*, as well as a large in-frame deletion in an isolate from one CFS subject and a premature stop codon was detected in an isolate from one healthy subject. These results suggest that the source of these sequences was diverse in nature and clearly not clonal or isogenic.

**Table 2:** **XMRV gag amino acid residues 8 to 120 in CFS**, **CFS positive control and healthy subjects**

**XMRV gag codon**		**024**	**031**	**032**	**034**	**035**	**036**	**045**	**046**	**047**	**048**	**049**	**050**	**051**	**059**	**060**	**105**
**Sample type**	**Sample ID**																
Published reference	VP62	S	K	K	R	W	V	T	F	N	V	G	W	P	G	V	P
Positive control reference	22Rv1	.	.	.	.	.	.	.	.	.	.	.	.	.	.	I	.
CFS positive control subject	G018	.	R	.	.	.	I								S	I	L
CFS subject	G026	.	R	.	.	.	I	.	.	.	.	.	.	.	S	I	L
CFS subject	G028	.	R	.	.	.	I	.	F/S	.	.	.	.	.	S	I	L
CFS subject	G035	.	R	.	.	.	I	.	.	.	.	.	.	.	S	I	L
CFS subject	G071	.	G	.	.	.	V	—	—	—	—	—	—	—	.	I	L
CFS subject	G091	.	R	.	R/C	.	I	.	.	.	.	.	.	.	S	I	L
Healthy subject	G053	.	R	.	.	.	I	.	.	.	.	.	.	.	S	I	L
Healthy subject	G046	P	R	K/E	.	Stop											

Noted along the top are XMRV Gag amino acid residue positions where a difference was observed in one or more subject samples relative to the VP62 reference virus sequence. Single letter amino acid abbreviations are given for the VP62 reference virus. XMRV gag amino acid residues 8 to 120 in the 22Rv1 positive control reference virus, one CFS Positive Control Subject, five previously untested CFS Subjects and two Healthy Subjects are displayed. Residues that are identical to the VP62 reference are denoted by a dot (.), differences are denoted by a single letter amino acid abbreviation, and deletions are denoted by a dash (—). Healthy Subject G046 has a premature stop codon at residue 35. Also of note, Healthy Subject G053 has a single nucleotide deletion upstream of the Gag start codon (not shown).

**Figure 1:**
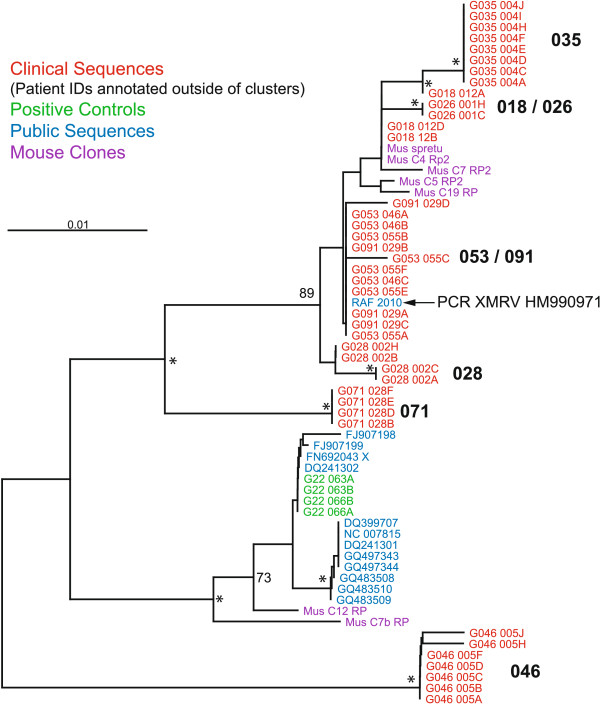
**Phylogenetic tree of murine retroviral DNA sequences from CFS and healthy subjects.** Results are displayed for one CFS Positive Control Subject (G018), five CFS Subjects (G026, G028, G035, G071 and G091) and two Healthy Subjects (G046 & G053). For these subjects, the first four letters/digits are subject anonymous identifier numbers while the last four letters/digits identifies replicates. Public MLV (blue text) and top mouse clone hits, which are also retroviral homologs (purple text), are included in the tree reconstruction. The position of the previously reported, PCR XMRV contaminant sequence (HM990971) is indicated [29]. The tree was reconstructed by neighbor-joining (NJ) method using DNA distance matrices of core conserved nucleotides (see Methods). Asterisks (“*”) indicate those nodes supported 70% or greater in 1000 bootstrap replicate NJ trees and 0.95 Bayesian posterior probability while nodes with numbers had high NJ bootstrap values only. Scale bar represents 0.1 expected nucleotide substitutions per site.

To further characterize these sequences, a phylogenetic analysis was performed (Figure 1) that included nucleotide sequences from 7 endogenous, murine retroviral and 13 other related reference strains. The major nodes in the phylogenetic tree were well-supported by two tree reconstruction methods, neighbor-joining (NJ) bootstrap and Bayesian analyses. Strong bootstrap and Bayesian probability support for clusters of replicates from individual subjects was also observed suggesting that the clones were derived from a common founder event that was distinct to each subject. All subject amplicons were more closely related to known clones of murine, endogenous retroviral sequences (nucleotide diversity as the average number of net nucleotide substitutions per site between populations, *d*_*A*_ = 0.02201 ± 0.0) than to previously reported XMRV or pMLV sequences (*d*_*A*_ = 0.0386 ± 0. 0.009) [28]. Nearly all sequences from subjects GO53 and G091 were 100% identical to HM990971, which was previously reported as a contaminant [29]. Among subject sequences from this study, nucleotide diversity was slightly higher (π = 0.0057 ± 0.0006) than among previously published XMRV and pMLV sequences (π = 0.00206 ± 0.00063), suggesting greater sequence variation across our sample set.

Interestingly, repeat testing of the apparent positives did not result in the detection of additional, PCR positive results. Fresh blood redraws from these subjects also did not result in the detection of any additional murine retroviral DNA by the Lo Method. Collectively, these results suggest that the detected sequences exist at a low frequency that is not robustly detected by the Lo Method.

To determine if the samples and/or reagents harbored murine DNA, a previously described intracisternal-A particle (IAP) [30,31] detection assay was employed [15]. The sensitivity of this assay was determined with a molecular clone, pIAP, which was engineered with a 71 base pair target sequence specific for the primers and probe and derived from the mouse genome but modified so that a false negative that may arise from carry over from the pIAP positive control could be resolved from the wild-type sequence (see Methods). The LLOD for this method was determined by serial dilution of pIAP in a background 50 ng of sheared salmon sperm DNA (an unlikely source of murine genomic DNA) and was observed to be 6 IAP copies per reaction. Whereas no IAP sequences were detected in the clinical isolates from which murine retroviral DNA was detected by the Lo Method, low level IAP DNA (~60 IAP copies in 9 of 9 replicates) was detected in the Lo Method PCR reagents employed in this study (data not shown). This observation is similar to the findings of others [15,29,32-34].

## Discussion

Many efforts to confirm the original claim by Lombardi *et al.*[1] of an association between the detection of XMRV and CFS have failed [3-24]. However, one report detected pMLV, instead of XMRV [2]. In an effort to confirm the original study and to better understand these collective, discrepant results, we hypothesized that the ability to detect these viruses not only depended upon the technical details of the methods employed but also on the criteria used to diagnose CFS and the availability of well characterized clinical isolates. We elected to compare and attempt to validate the published methods in a prospective study with fresh, well defined clinical isolates. The establishment of the SCB not only provided fresh, well characterized and geographically distinct samples for our blinded, prospective study, but also made available isolates that could serve as positive controls for assay validation efforts.

With these fresh samples, we were able to detect murine, retroviral signatures, however the collective overall incidence of detection in samples from the validation and blinded phases of this study was low (5-6.9%). This inability to robustly detect these sequences was surprising since the reported incidence was so high [1,2] and the method employed proved to be highly sensitive in our hands. The reason for this discrepancy is even less clear as some reports suggest that the source of the detected retroviral sequences may originate from contamination of the PCR reagents with murine genomic DNA [15,29]. If this were the source of the observed murine retroviral sequences, then one might expect that the frequency of detection would be higher for such a sensitive assay. However, we could not detect these murine, retroviral signatures in repeat testing of the same initial samples after the study was unblinded. We also could not confirm the detection of murine retroviruses in isolates from repeat blood draws from subjects in which these sequences were initially observed in the blinded phase of this study. Collectively, these results suggest that the incidence of these detected sequences must be very low; likely near the LLOD of the assay.

While we did not test for the presence of murine retrovirus-related antibodies in these subjects, the nature of the observed sequences appeared to be consistent with a murine genomic DNA source as evidenced by direct comparisons at the coding level (Table 2) as well as a phylogenetic analysis at the nucleotide level (Figure 1). The observed greater inter- versus intra-subject viral sequence diversity is consistent with a murine genomic source and not an individual founder virus or isogenic, nucleic acid-based control. This presumption was confirmed by testing for IAP DNA which was observed in the PCR reagents but not the clinical isolates. We carefully quantitated the amount of this material at ~30 IAP copies per unit of Taq DNA polymerase which is much less than 1 murine cell genomic equivalent (~2,000 IAP copies/cell). It is possible that the amount of this material varies greatly from batch to batch and that the preparations employed in the original studies harbored a much higher murine genomic equivalent [31]. However, it still remains unclear as to why the previously, published studies did not observe an equivalent incidence of these sequences in isolates from CFS and healthy subjects [1,2].

Although our study inclusion criteria were quite rigorous, subject demographics and sample selection criteria represent potential constraints for the interpretation of results from this study. Since a diagnosis of CFS requires clinical evaluation, this study was designed to work with clinicians with expertise in CFS diagnosis and management. However, it is known that patients in tertiary care clinics, such as those tested in this study, are not strictly representative of the general CFS patient population and are more likely to be Caucasian, female, and more severely ill [35]. Despite this, the results of this study are consistent with those of another prospective confirmation effort [19] and the SCB should prove to be a valuable resource for the discovery of CFS-related biomarkers and treatments in future studies.

## Conclusions

The results of this blinded, multi-site, prospective study are consistent with those of other confirmation studies. Specifically, using previously published PCR-based detection methods, we found no difference in the incidence of detectable murine retroviral sequences in the PBMC DNA from well characterized CFS and healthy subjects. In addition, our results suggest that the Taq DNA polymerase that was used in the PCR detection method was the source of the murine retroviral signatures observed in isolates from both the CFS and healthy subjects tested in this study. CFS represents a major unmet medical need and additional studies are needed to determine the etiology of the syndrome so that effective treatments may be discovered.

## Methods

### Establishment of the “SolveCFS Biobank” clinical repository

The initial seeding of the SCB clinical repository, led by the CFIDS Association of America, was achieved by the collection of blood samples from a total of 240 CFS and 87 healthy subjects in three months (June to August 2010). Subjects were selected from five, geographically distinct clinical sites that specialized in the diagnosis and management of CFS. Of these, 83 CFS and 55 healthy subjects were enrolled by a Utah-based clinic, 74 CFS and 8 healthy subjects by a clinic in North Carolina, 43 CFS and 24 healthy subjects by a Florida-based clinic, 1 CFS subject by a clinic in Pennsylvania and 40 CFS subjects who had tested positive for XMRV prior to this study (CFS Positive Controls) by a Nevada-based clinical site. The latter were intended to serve as clinical positive controls for use in detection method validation. The 87 healthy subject samples collected were from individuals who were geographically co-localized (same neighborhood or region; but not residing in the same household or of close relation to a CFS subject) and were matched to the CFS subjects by age (within 5 years), sex, and race. After receipt of informed consent, the SCB coordinator assigned each participant a unique identifier and arranged for the completion of a questionnaire by mail aimed at resolving details relevant for the consideration of inclusion in the study.

### Sample collection, processing and storage

Each clinical site was provided with blood collection kits that included four, 10 mL sodium-heparin, Vacutainer™ tubes (Becton Dickinson; Franklin Lakes, NJ) and return shipping materials by the SCB. The collection and processing methods were similar to those defined in the study by Lombardi *et al*. 2009. Blood was collected into the SCB-provided collection tubes by venipuncture using standard phlebotomy procedures and shipped overnight by express mail at ambient temperature to the RUCDR for processing. PBMCs were processed by the standard operating procedures of the RUCDR. The collection tubes were centrifuged at 1,200 × g at room temperature to separate plasma and cells. After collection of plasma, the remaining PBMC band was purified by density gradient centrifugation at 800 × g for 22 minutes after layering on NycoPrep™ (Axis Shield, PoC, Oslo, Norway). Purified PBMCs were aspirated and washed once with Dulbecco’s phosphate-buffered saline (Life Technologies, Carlsbad, CA). Dry and TRIzol (Life Technologies; Carlsbad, CA)-resuspended cell pellets of 10 million cells each were prepared by centrifugation at 500 × g for 7 minutes and stored at -80°C. An additional 10 million cells were cryopreserved in 90% FBS and 10% DMSO and stored in liquid nitrogen. Dry and TRIzol cell pellets were shipped to GSK for DNA and RNA isolation and subsequent viral detection methods. For all subjects with a positive result for the detection of MLV-related sequences, a subsequent separate blood sample was collected and dry and TRIzol cell pellets were prepared and shipped to GSK as described above.

### Subject population

A CFS subject was eligible for inclusion in this study if they had previously been diagnosed with CFS by either the Fukuda or the Canadian criteria [36,37], in addition to having initial presentation of flu-like illness or an acute (48 hours) or subacute (4 weeks) onset; fatigue that persisted for at least six months; post-exertional malaise lasting >24 hours; significant cognitive impairment in short-term memory and concentration; RAND-36 quality of life survey [38-40] results that meet 2 of the 3 following benchmarks: vitality <35, social functioning <62.5, role-physical <50; and age between 18 and 65 years at the time of signing the informed consent.

Female subjects were eligible to participate if not pregnant, not <3 months postpartum, and not currently lactating per self-report. A subject was excluded if they had a body mass index >40, an immunosuppressive disorder including, but not limited to cancer, severe infections or HIV. In addition, subjects were excluded if they had a history of substance or alcohol abuse < 2 years before onset of CFS or were mentally or legally incapacitated at the time of collection. De-identified registry data was provided to GSK for verification prior to receipt of isolates for study.

### Isolation of nucleic acid from clinical isolates

DNA was extracted and purified from dry cell pellets by silica-based adsorption with a QIAamp DNA Blood Mini Kit (Qiagen; Valencia, CA). RNA was prepared from separate cell pellets by guanidinium thiocyanate-phenol-chloroform extraction using the TRIzol reagent by Life Technologies (Carlsbad, CA). Yield and purity for nucleic acid preparations was determined by UV spectroscopy with a NanoDrop spectrophotometer (NanoDrop Products; Wilmington, DE).

### Positive controls for PCR detection methods

A positive control for XMRV was created by TOPO TA cloning (Life Technologies, Carlsbad CA) PCR amplified XMRV gag DNA products from 22RV1 cells using the previously described 419F and 1154R primers. Plasmid DNA was purified with a PureLink HiPure Plasmid Maxiprep Kit (Life Technologies; Carlsbad, CA) and quantified by UV spectroscopy with a NanoDrop Spectrophotometer. The cloned *gag* insert was sequence verified and found to be identical to XMRV-VP62 (GenBank accession number EF185282.1) with the exception of a guanine to adenine substitution at position 367 relative to the 5′ end of the 419F primer. An IAP containing plasmid, pIAP, was created in a pUC57 backbone by GenScript (Piscataway, NJ). The 71 base pair IAP sequence (5′-GCCGC GCCCA CATTC GCCGT TACAA GATGG TGCTG AC*TCG ACA*GT TCTAA GTGGT AAACA AATAA TCTGC G-3′), was derived from the murine genome and designed to be recognized by the primers and probe from a previously described method. To enable the resolution of false from real IAP-positive PCR detection, 6 nucleotides situated between the probe and downstream primer sequence were changed to their complementary base (italicized). The resulting sequence is not 100% homologous to any known sequence of the mouse genome and, as such, would be easily resolved by sequencing. This would allow resolution of whether an IAP positive sample was contaminated with material of mouse origin or from the assay positive control.

### PCR-Based XMRV and IAP detection assays

PCR primers were synthesized by Integrated DNA Technologies (Coralville, IA). For the XMRV DNA detection method described by Lombardi *et al*., 200 ng of subject DNA (~33,000 cell equivalents) was amplified using the gag 419F and 1154R primers as described [1]. For the XMRV DNA detection method described by Lo *et al*. (Lo Method), 50 ng of subject DNA (~8,250 cell equivalents) was employed and tested as described [2]. The IAP assay was as described by Shin et al. [15]. For all PCR-based detection methods, products were resolved by agarose gel electrophoresis using 15 μL of the PCR reaction. PCR products that migrated in the gel to a size that was within approximately 150 base pairs of each assay’s respective positive control were extracted from the gel, purified using the MinElute Gel Extraction Kit (Qiagen; Valencia, CA) and cloned with a TOPO TA Cloning Kit (Life Technologies; Carlsbad, CA). Cloned DNA was purified with the PureLink Quick Plasmid Miniprep Kit (Life Technologies; Carlsbad, CA), and sequenced by Sanger chain-terminating dideoxynucleotide sequencing (Life Technologies; Carlsbad, CA). If at least 1 clone was identified with >95% sequence identity by BLASTN [41] searches of GenBank (Nonredundant or nr database version February 2011) to published XMRV or MLV-related sequences, the individual was considered positive for harboring XMRV/MLV-related viral DNA. DNA sequences are available from NCBI (GenBank Accession# KM222449 -> KM222492).

### Statistical analyses

Demographics as well as Physical and Mental Health data (Table 1) were analyzed using SAS 9.2 (SAS Institute, Cary, NC). Figures were created in JMP 11.0 (SAS Institute, Cary, NC). We were interested in the following comparisons: CFS Subjects vs. Healthy Subjects, CFS Subjects vs. CFS Positive Control Subjects, and Healthy Subjects vs. CFS Positive Control Subjects. Comparing XMRV Positive versus XMRV Negative Subjects within or between groups was not feasible due sample size limitations. The continuity-corrected Wilcoxon-Mann-Whitney test was used for all continuous variables. The normal approximations statistic Z and the two sided p-values are provided in the Additional file 1: Table S1. Fisher’s exact test was used for all categorical variables, and the results are provided in Additional file 2: Table S2. Missing data were not included in the statistical analyses.

### Phylogenetic analysis

Initial multiple sequence alignments of the conserved region of the *gag* gene were performed using the program CLUSTALW v1.83 [42] with default settings and subsequently, refined manually using the program SEQLAB of the GCG Wisconsin Package v11.0 software package (Accelrys, San Diego, CA, USA). A final alignment 339 nucleotides in length was used for all subsequent phylogenetic analyses. We constructed phylogenetic trees using distance NJ and Bayesian posterior probabilities (BP). NJ trees were based on pair wise distances between nucleic acid sequences using the programs NEIGHBOR and DNADIST (Dayhoff option) of the PHYLIP 3.6 package [43]. The programs SEQBOOT and CONSENSE were used to estimate the confidence limits of branching points from 1000 bootstrap replications. BP trees were constructed using the software MrBayes v3.0B4 [44,45]. The gamma-distributed rate model with 6 discrete rate categories (GTR model) was chosen. Markov chains were run for 10^6^ generations, burn-in values were set for 10^4^ generations, and trees sampled every 100 generations. All trees were visualized using the program TREEVIEW v1.6.6 [46]. Nucleotide diversity ± standard deviation within populations (π) and average number of net nucleotide substitutions per site between populations (*d*_*A*_) was calculated using the software DnaSPv5 [47].

### Ethical approval

The subjects who participated in this study gave informed consent and the protocol and the procedures of the study were conducted in conformity with the ethical guidelines of the Declaration of Helsinki. The protocol for laboratory research, including analysis of blood samples and clinical information, was reviewed and approved by Copernicus Group independent review board (IRB# GLA1-10-102). The collection of clinical information and biological samples by the SolveCFS BioBank was ethically approved by the Genetic Alliance ethics review board (IRB # IORG0003358).

## Abbreviations

XMRV: Xenotropic murine leukemia virus-related virus; CFS: Chronic fatigue syndrome; PCR: Polymerase Chain Reaction; pMLV: polytropic murine leukemia virus; MLV: Murine leukemia virus; GSK: GlaxoSmithKline; CFIDS: Chronic Fatigue and Immune Dysfunction Syndrome; SCB: SolveCFS BioBank; RAND-36: RAND-36 health-related quality of life questionnaire; RUCDR: Rutgers University Cell and DNA Repository; LLOD: Lower limit of detection; PBMCs: peripheral blood mononuclear cells; IAP: Intracisternal A-type particle; NJ: Neighbor-joining; BP: Bayesian posterior probabilities.

## Competing interests

The authors declare that they have no competing interests.

## Authors’ contributions

DAW, PG and SDV designed the study. SDV and KKM established the SolveCFS BioBank. DP, LB, NGK, and WCL collected the samples. DMI and PG performed the testing and data analysis. JRB performed the phylogenetic analyses. KSJ performed the statistical analyses. DMI, PG, DAW, JRB and SDV wrote the manuscript. All authors read and approved the manuscript.

## Supplementary Material

Additional file 1: Table S1 Statistical Comparisons of CFS, Healthy and CFS Positive Control Subjects for Demographics, Physical and Mental Health. Demographics, physical and mental health data were analyzed comparing CFS Subjects vs. Healthy Subjects, CFS Subjects vs. CFS Positive Control Subjects, and Healthy Subjects vs. CFS Positive Control Subjects. The continuity-corrected Wilcoxon-Mann-Whitney test was used for all continuous variables. The normal approximations statistic Z and the two sided p-values are provided for each comparison. CFS Subjects and CFS Positive Control Subjects exhibited significantly lower physical and mental health scores when compared to those reported by the Healthy Subjects. Click here for file

Additional file 2: Table S2 Statistical Comparisons of CFS, CFS Positive Control and Healthy Subjects for Sex, Race, CFS Onset and XMRV Status. Fisher’s exact test results comparing CFS vs. Healthy Subjects, CFS vs. CFS Positive Control Subjects, and Healthy vs. CFS Positive Control Subjects for Sex, Race, CFS Onset and XMRV Status. No significant difference in the prevalence of murine, retroviral nucleic acid signatures (XMRV Positive and Negative Status) was observed between the CFS and Healthy Subject groups. Click here for file

Additional file 3: Figure S1 Physical and Mental Health and Demographics for CFS, CFS Positive Control and Healthy Subjects. Scatter plots of a. RAND-36 physical health scores, b. RAND-36 mental health scores and c. demographics of CFS Subjects, Healthy Subjects and CFS Positive Control Subjects. Subjects with a positive test result for XMRV/murine retroviral sequences as a result of testing in this report are indicated by a cross (x) and subjects with a negative XMRV test result are indicated by a filled circle (●). Mean values for each subject group are indicated by a dash (–). Click here for file
