# Supplementary material for: No association found between the detection of either xenotropic murine leukemia virus-related virus or polytropic murine leukemia virus and chronic fatigue syndrome in a blinded, multi-site, prospective study by the establishment and use of the SolveCFS BioBank
Source: BMC Res Notes. 2014 Aug 4;7:461. doi: 10.1186/1756-0500-7-461 (PMC4236736; doi:10.1186/1756-0500-7-461)
Supplement: Additional file 1: Table S1 — Statistical Comparisons of CFS, Healthy and CFS Positive Control Subjects for Demographics, Physical and Mental Health. Demographics, physical and mental health data were analyzed comparing CFS Subjects vs. Healthy Subjects, CFS Subjects vs. CFS Positive Control Subjects, and Healthy Subjects vs. CFS Positive Control Subjects. The continuity-corrected Wilcoxon-Mann-Whitney test was used for all continuous variables. The normal approximations statistic Z and the two sided p-values are provided for each comparison. CFS Subjects and CFS Positive Control Subjects exhibited significantly lower physical and mental health scores when compared to those reported by the Healthy Subjects. [file 1756-0500-7-461-S1.docx]

**Supplementary Table 1. Statistical Comparisons of CFS, Healthy and CFS Positive Control Subjects for Demographics, Physical and Mental Health**

Demographics, physical and mental health data were analyzed comparing CFS Subjects vs. Healthy Subjects, CFS Subjects vs. CFS Positive Control Subjects, and Healthy Subjects vs. CFS Positive Control Subjects. The continuity-corrected Wilcoxon-Mann-Whitney test was used for all continuous variables. The normal approximations statistic Z and the two sided p-values are provided for each comparison. CFS Subjects and CFS Positive Control Subjects exhibited significantly lower physical and mental health scores when compared to those reported by the Healthy Subjects.

| **Type** | **Response** | **Comparison** | **Z Statistic** | **p-value (Z)** |
| --- | --- | --- | --- | --- |
| Demographics | Age | CFS Subjects vs. Healthy Subjects | -0.8974 | 0.3695 |
|  |  | CFS Subjects vs. CFS Positive Control Subjects | 0.3544 | 0.723 |
|  |  | Healthy Subjects vs. CFS Positive Control Subjects | 0.82 | 0.4122 |
|  | Age of first CFS Symptoms | CFS Subjects vs. CFS Positive Control Subjects | -0.2813 | 0.7785 |
|  | Age of CFS Diagnosis | CFS Subjects vs. CFS Positive Control Subjects | -0.8723 | 0.3831 |
|  | BMI | CFS Subjects vs. Healthy Subjects | -0.5034 | 0.6147 |
|  |  | CFS Subjects vs. CFS Positive Control Subjects | -0.9536 | 0.3403 |
|  |  | Healthy Subjects vs. CFS Positive Control Subjects | -0.3973 | 0.6912 |
| RAND-36 Physical Health | Physical Functioning | CFS Subjects vs. Healthy Subjects | 6.7673 | <.0001 |
|  |  | CFS Subjects vs. CFS Positive Control Subjects | 0.3413 | 0.7328 |
|  |  | Healthy Subjects vs. CFS Positive Control Subjects | -4.9063 | <.0001 |
|  | Role Physical | CFS Subjects vs. Healthy Subjects | 8.9103 | <.0001 |
|  |  | CFS Subjects vs. CFS Positive Control Subjects | -0.5188 | 0.6039 |
|  |  | Healthy Subjects vs. CFS Positive Control Subjects | -6.3919 | <.0001 |
|  | Bodily Pain | CFS Subjects vs. Healthy Subjects | 6.1001 | <.0001 |
|  |  | CFS Subjects vs. CFS Positive Control Subjects | -0.4707 | 0.6378 |
|  |  | Healthy Subjects vs. CFS Positive Control Subjects | -5.0094 | <.0001 |
|  | General Health | CFS Subjects vs. Healthy Subjects | 7.5217 | <.0001 |
|  |  | CFS Subjects vs. CFS Positive Control Subjects | -2.7684 | 0.0056 |
|  |  | Healthy Subjects vs. CFS Positive Control Subjects | -5.7294 | <.0001 |
| RAND-36 Mental Health | Vitality | CFS Subjects vs. Healthy Subjects | 7.1449 | <.0001 |
|  |  | CFS Subjects vs. CFS Positive Control Subjects | 0.2575 | 0.7968 |
|  |  | Healthy Subjects vs. CFS Positive Control Subjects | -5.1756 | <.0001 |
|  | Social Functioning | CFS Subjects vs. Healthy Subjects | 7.3295 | <.0001 |
|  |  | CFS Subjects vs. CFS Positive Control Subjects | -0.7748 | 0.4384 |
|  |  | Healthy Subjects vs. CFS Positive Control Subjects | -5.4676 | <.0001 |
|  | Role Emotional | CFS Subjects vs. Healthy Subjects | 1.8385 | 0.066 |
|  |  | CFS Subjects vs. CFS Positive Control Subjects | -1.1398 | 0.2544 |
|  |  | Healthy Subjects vs. CFS Positive Control Subjects | -2.6481 | 0.0081 |
|  | Mental Health | CFS Subjects vs. Healthy Subjects | 4.7719 | <.0001 |
|  |  | CFS Subjects vs. CFS Positive Control Subjects | -0.7885 | 0.4304 |
|  |  | Healthy Subjects vs. CFS Positive Control Subjects | -3.7774 | 0.0002 |
