# Supplementary material for: No association found between the detection of either xenotropic murine leukemia virus-related virus or polytropic murine leukemia virus and chronic fatigue syndrome in a blinded, multi-site, prospective study by the establishment and use of the SolveCFS BioBank
Source: BMC Res Notes. 2014 Aug 4;7:461. doi: 10.1186/1756-0500-7-461 (PMC4236736; doi:10.1186/1756-0500-7-461)
Supplement: Additional file 2: Table S2 — Statistical Comparisons of CFS, CFS Positive Control and Healthy Subjects for Sex, Race, CFS Onset and XMRV Status. Fisher’s exact test results comparing CFS vs. Healthy Subjects, CFS vs. CFS Positive Control Subjects, and Healthy vs. CFS Positive Control Subjects for Sex, Race, CFS Onset and XMRV Status. No significant difference in the prevalence of murine, retroviral nucleic acid signatures (XMRV Positive and Negative Status) was observed between the CFS and Healthy Subject groups. [file 1756-0500-7-461-S2.docx]

**Supplementary Table 2. Statistical Comparisons of CFS, CFS Positive Control and Healthy Subjects for Sex, Race, CFS Onset and XMRV Status**

Fisher’s exact test results comparing CFS vs. Healthy Subjects, CFS vs. CFS Positive Control Subjects, and Healthy vs. CFS Positive Control Subjects for Sex, Race, CFS Onset and XMRV Status. No significant difference in the prevalence of murine, retroviral nucleic acid signatures (XMRV Positive and Negative Status) was observed between the CFS and Healthy Subject groups.

| **Response** | **Comparison** | **p-value (Fishers's Exact Test)** |
| --- | --- | --- |
| Sex | CFS Subjects vs. Healthy Subjects | 0.44 |
|  | CFS Subjects vs. CFS Positive Control Subjects | 0.1327 |
|  | Healthy Subjects vs. CFS Positive Control Subjects | 0.0382 |
| Race | CFS Subjects vs. CFS Positive Control Subjects | 0.1932 |
|  | Healthy Subjects vs. CFS Positive Control Subjects | 0.3269 |
| CFS Onset | CFS Subjects vs. CFS Positive Control Subjects | 0.1362 |
| XMRV Positive and Negative Status | CFS Subjects vs. Healthy Subjects | 1 |
|  | CFS Subjects vs. CFS Positive Control Subjects | 1 |
|  | Healthy Subjects vs. CFS Positive Control Subjects | 1 |
