# Supplementary material for: No association found between the detection of either xenotropic murine leukemia virus-related virus or polytropic murine leukemia virus and chronic fatigue syndrome in a blinded, multi-site, prospective study by the establishment and use of the SolveCFS BioBank
Source: BMC Res Notes. 2014 Aug 4;7:461. doi: 10.1186/1756-0500-7-461 (PMC4236736; doi:10.1186/1756-0500-7-461)

Supplementary Figure 1. Physical and Mental Health and Demographics for CFS, CFS Positive Control and Healthy Subjects

Scatter plots of a. RAND-36 physical health scores, b. RAND-36 mental health scores and c. demographics of CFS Subjects, Healthy Subjects and CFS Positive Control Subjects. Subjects with a positive test result for XMRV/murine retroviral sequences as a result of testing in this report are indicated by a cross (x) and subjects with a negative XMRV test result are indicated by a filled circle (●). Mean values for each subject group are indicated by a dash (–).

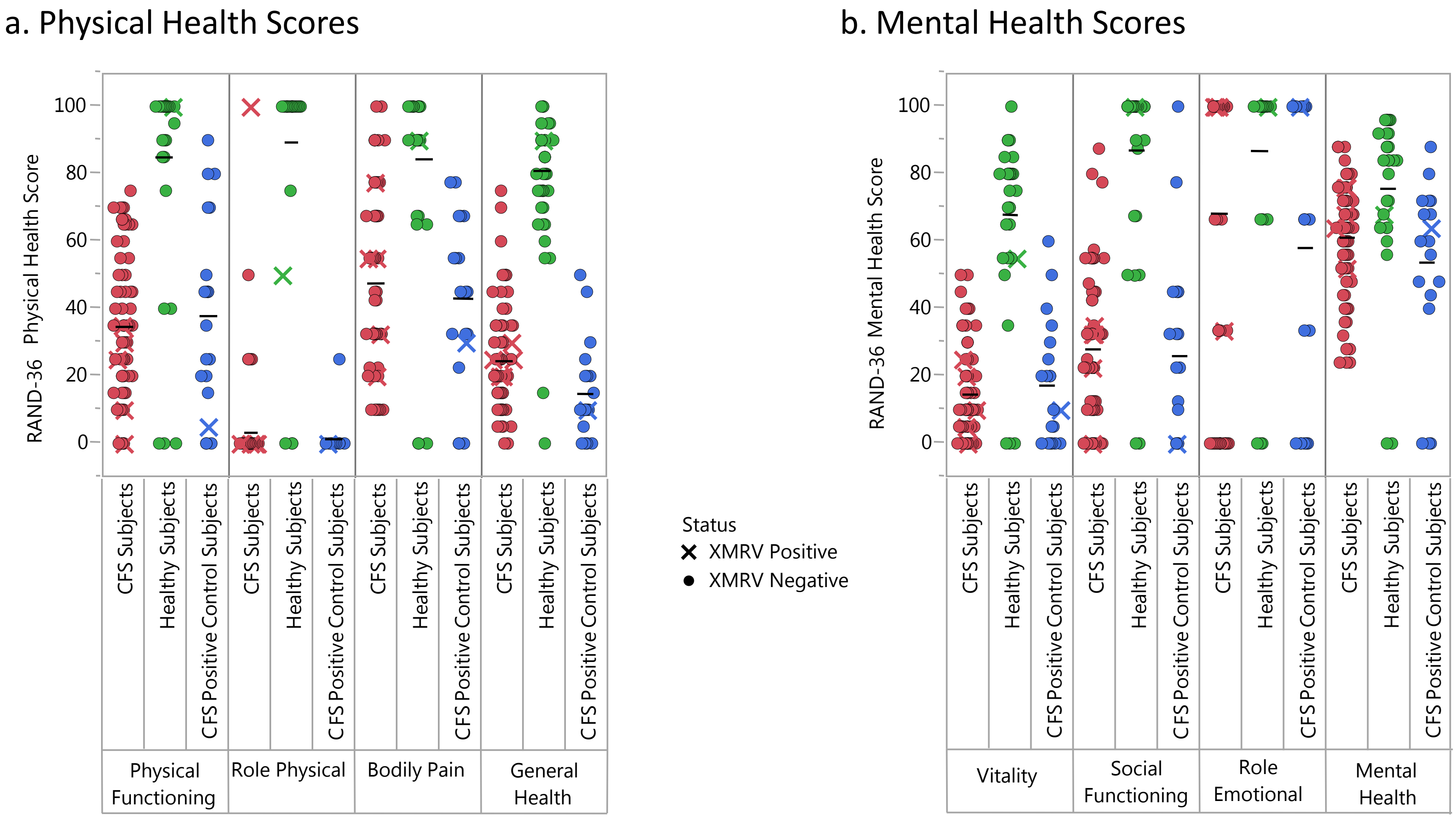

c. Demographics

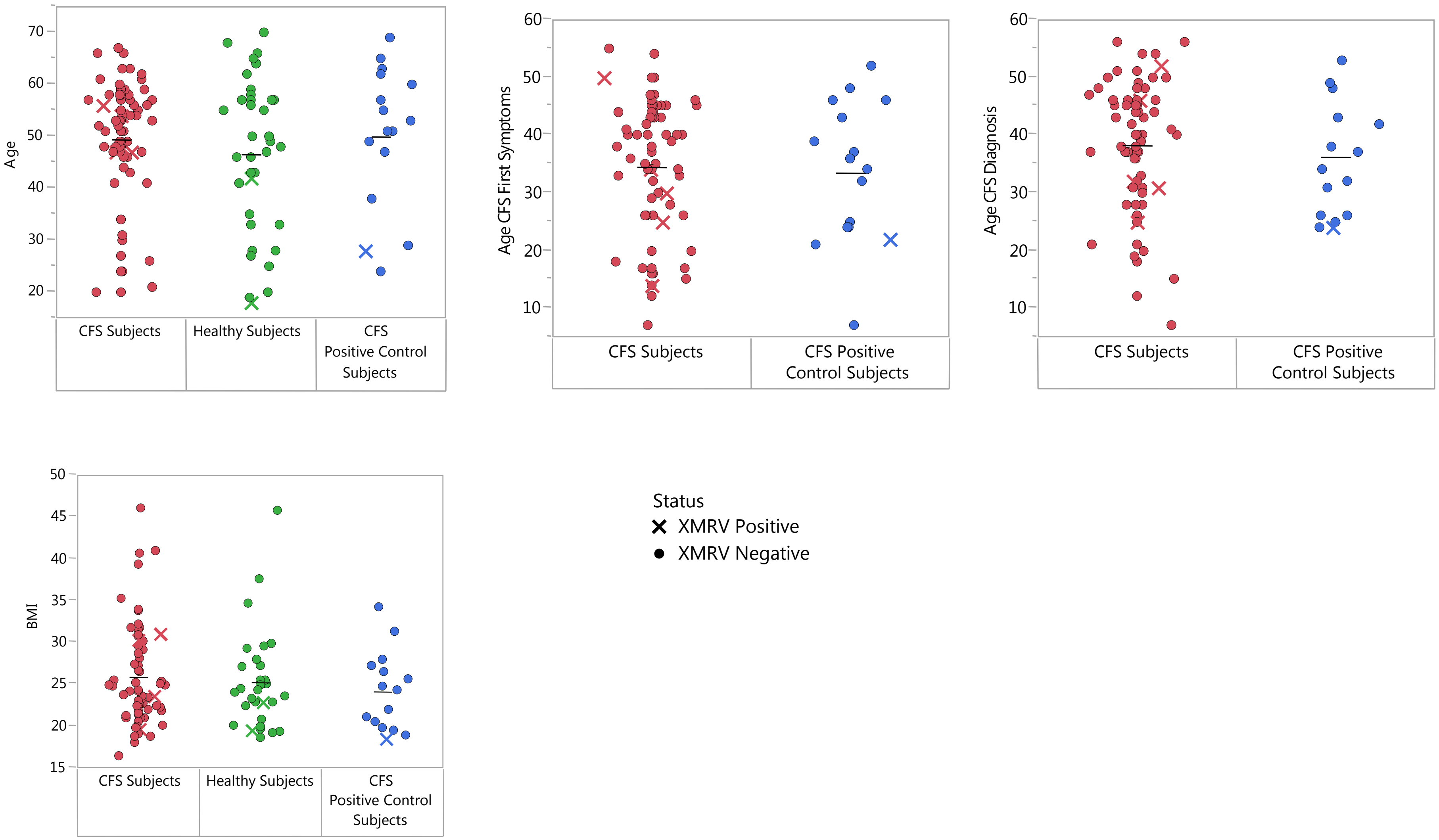

Supplement: Additional file 3: Figure S1 — Physical and Mental Health and Demographics for CFS, CFS Positive Control and Healthy Subjects. Scatter plots of a. RAND-36 physical health scores, b. RAND-36 mental health scores and c. demographics of CFS Subjects, Healthy Subjects and CFS Positive Control Subjects. Subjects with a positive test result for XMRV/murine retroviral sequences as a result of testing in this report are indicated by a cross (x) and subjects with a negative XMRV test result are indicated by a filled circle (●). Mean values for each subject group are indicated by a dash (–). [file 1756-0500-7-461-S3.pdf]
